# Anaplastic thyroid cancer: multimodal treatment results

**DOI:** 10.3332/ecancer.2014.449

**Published:** 2014-07-30

**Authors:** Zaki Antonio Taissoun Aslan, Martín Granados-García, Kuauhyama Luna-Ortiz, Francisco Javier Guerrero-Huerta, Antonio Gómez-Pedraza, Silvio A Ñamendys-Silva, Abelardo Meneses-García, Juliana María Ordoñez-Mosquera

**Affiliations:** 1 Department of Head and Neck, Instituto Nacional de Cancerología, Mexico; 2 Surgical Subdivision, Instituto Nacional de Cancerología, Mexico; 3 Department of Pathology, Instituto Nacional de Cancerología, Mexico; 4 Department of Surgery, Universidad Militar UMNG de Colombia, Bogotá DC

**Keywords:** anaplastic thyroid cancer, multimodal treatment, prognoses, thyroid cancer

## Abstract

**Background:**

Anaplastic thyroid cancer is a rare and lethal disease. It accounts for 1–2% of thyroid malignancies, but specific mortality is higher than 90%. It is an aggressive locoregional disease with a high metastatic capacity. There is no agreement with regards to the best treatment. We analysed the results of treatment in a mestizo population treated in the National Cancer Institute (Mexico).

**Methods:**

We reviewed 1,581 files of thyroid carcinomas; of these, 29 (1.83%) had anaplastic thyroid carcinoma. Demographic variables, clinical manifestations, tumour characteristics, and treatments were analysed.

**Results:**

The median age was 64.5 ± 13.2 years. Females were more affected (female/male ratio: 2.6:1); 21 cases occurred in women (72.4%), and eight in males (27.6%). The most common manifestations were neck enlargement (93.10%) and hoarseness (71.31%). The median tumour size was 8 cm (range: 4–20 cm). The percentage of cases which presented in clinical stage IVA was 10.3%, with 62.1% presenting in clinical stage IVb and 27.6% presenting in clinical stage VIc. Complete resection (R0) (*p* = 0.05), radiation doses of higher than 33.1 Gy (*p* = 0.04), and multimodal therapy were associated with better survival. Surgery plus radiotherapy with or without systemic treatment (*p* = 0.006). The median overall survival was 119 days (IC 95%, 36.3–201.6). Six-month, one-year and two-year survival was 37.9%, 21% and 13%, respectively.

**Conclusion:**

Complete surgical resection is associated with better survival but is very difficult to achieve due to aggressive biological behaviour. Multimodal therapy is associated with better survival and a better quality of life. There is a need for more effective systemic treatments as extensive surgical resections have little overall benefit in highly invasive and metastatic disease.

## Introduction

Anaplastic thyroid cancer (ATC) is a defined pathological entity found among the undifferentiated neoplasms of the follicular epithelium of the thyroid gland. Fortunately, it is rare, with incidences of one to two cases per 1,000 inhabitants in areas with endemic goiter [1], and 1–2% of the malignant thyroid neoplasms in the United States [2]. Similar incidences reported in Norway represent 2.1% of thyroid neoplasms [3]. It is interesting to observe that the incidence rate of ATC has not varied despite the significant and recent increase in the diagnosis of malignant thyroid lesions other than ATC^4^.

It is a very lethal entity. 90% of cases occur in individuals in their 60s and 70s [5–8]. The female/male ratio is 1.5–2:1 [6, 7]. Its natural progression is overwhelming. Once it is diagnosed, the average symptomatic progression is 1.5 months, 0–8 months in the majority of cases [8, 9]. It appears as a solid mass in the centre of the neck, associated with vocal cord paralysis and dysphonia in 77% of cases, dysphagia in 56%, and adenopathy in 54%. Upon initial diagnosis, 50% of patients have distant metastasis, another 25% develop it a bit later [2, 10]. Despite its rareness, it accounts for 14–39% of deaths from thyroid neoplasm, and it is virtually 100% [1] fatal—the median survival rate barely reaches three to five months [5, 6].

Due to its high fatality rate, combining surgery, teletherapy, chemotherapy, and targeted therapies is controversial since the results are poor. The purpose of the present study is to evaluate the impact of these treatments among a Mexican-Mestizo population.

## Materials and methods

Patients treated for ATC between January 1992 and December 2012 were identified from institutional registries. We studied patients with pathologically confirmed diagnoses. Histopathologic diagnosis was established on a morphological base, but to remove all doubt, we carried out immunohistochemical studies, such as thyroglobulin, calcitonin, and cytokeratin (CD15–CD30) to rule out other undifferentiated thyroid and lymphoma carcinomas, and undifferentiated medullary thyroid cancer. Demographic variables, clinical manifestations, tumour characteristics, and treatment were analysed.

### Statistical analysis

Descriptive statistics were used to summarise the data. If the distribution of the data was abnormal (determined through the Kolmogorov– Smirnov test), the numerical variables were expressed as mean ± standard deviation, or median and interquartile ranges. The nominal variables were expressed as percentages. The Student’s *t* (normal distribution) or the Mann-Whitney *U* (abnormal distribution) was used to compare the continuous variables. To analyse the qualitative variables, the X^2^ test or the Fisher’s exact test was used as appropriate. Survival curves were created using the Kaplan-Meier method. Survival was compared with the log-rank test). In all cases, *p* < 0.05 from a two-tailed test was considered significant. The statistical package SPSS 20.0 was used for descriptive and inferential statistics.

## Results

Out of 1,581 with malignant thyroids, 29 patients with anaplastic cancer were studied, which represented 1.83% of the malignant thyroids treated at the Instituto Nacional de Cancerologia.

### Demographic and clinical variables

The disease was more prevalent in females, 21 cases (72.4%), compared to males, eight cases (27.6%), a ratio of 2.6:1. The median age was 64.5 ± 13.2, with age limits of 35–91. The majority of patients, 23 (79.3%), had multiple clinical manifestations upon diagnosis (Table 1).

Histopathologic diagnosis was established through incisional biopsy on 16 patients (55.2%). Guided fine-needle biopsy initially estimated the diagnosis in ten patients (10.3%) shortly after confirmed with tissue samples, but the histological diagnosis was not established for three patients until analysis after surgery.

Computed tomography (CT) was the study of choice for evaluating the extent of the disease; however, it indicated a correct diagnosis in 28 patients (96.5%). One patient refused to undergo a CT. One patient (3.4%) also underwent magnetic resonance imaging of the brain to confirm the suspicion of brain metastasis.

The median tumour size was 8 cm, within a range of 4–20 cm. 18 (62.1%) patients had locoregional extrathyroid disease, eight (27.6%) had distant metastasis, and three (10.3%) had intrathyroid disease at the time of diagnosis.

The clinical stage distribution (AJCC, 2010) [11] can be seen in Table 2.

The development of distant metastasis was very common. At the time of diagnosis, there were eight patients who presented with it, and eight developed it shortly after, for a total of 16 (55.2%).

The metastases were: lungs, 14 patients (48.27%); brain, 1 (3.4%); liver, 1 (3.4%); and bones, 1 (3.4%). One patient presented with metastases in two different places.

### Treatment

13 out of 29 patients (44.8%) were considered non-resectable, due to the presence of extensive extrathyroid disease that affected the trachea, mediastinum vessels, cervical oesophagus, prevertebral fascia, or poor functional health.

A total thyroidectomy was performed on nine patients (31%); one patient received a laryngopharyngectomy with a complete resection of the tumour (R0). Two patients (6.8%) had a central and bilateral anterolateral neck dissection (levels II–VI) with resection of the spinal nerve and internal jugular vein. We achieved total resection (R0) in four (13.7%) patients.

A partial thyroidectomy was performed in four patients (13.7%). One received a bilateral radical neck dissection (levels I–V). All had macroscopic residual disease (R2), (Table 3).

An incisional biopsy was done on three patients (10.3%), after a failed resection.

It is possible to classify the patients into two groups. The first is characterised by incomplete resections or no resection, consisting of 25 patients (86.2%): four patients (13.8%) with microscopic residual tumour tissue (R1) and 21 patients (72.4%) with macroscopic residual tumour tissue (R2). The second group, made up of only four patients (13.8%), is characterised by total resections. Survival was associated with the quality of the resection.

**Figure 1.**
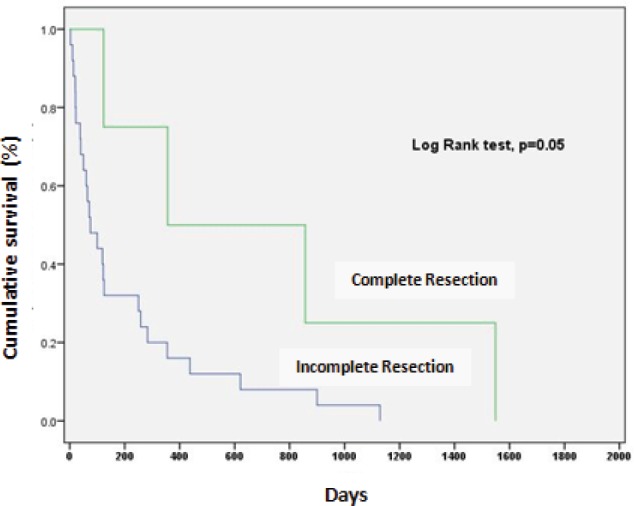
Survival according to quality of the surgical resection.

Complete resection (R0) was associated with significantly better survival in patients with residual tumour (*p* = 0.05) (Figure 1).

#### Adjunctive therapy

##### Chemotherapy and radiotherapy post-operative concomitant

After surgery, seven patients received concomitant radiotherapy with systemic therapy. We used doxorubicin only in small doses of 10–15 mg/m^2^ weekly on 11 patients (37.9%), doxorubicin 50 mg/m^2^ plus cisplatin 40 mg/m2 every three weeks on one patient (3.4%), and doxorubicin plus sorafenib 400 mg bid for a 28-day cycle on a patient with brain metastasis (3.4%).

##### Post-operative radiotherapy

Four patients received post-operative radiotherapy exclusively, two of them after a total thyroidectomy, and the other after a partial thyroidectomy.

#### Surgery only

Two patients were treated exclusively with surgery.

#### Definitive treatment without surgery

##### Chemotherapy and radiotherapy definitive concomitant

There were six patients treated with concomitant chemotherapy and radiotherapy without surgical resection, due to clear non-resectability or after an unsuccessful resection.

#### Radiotherapy only

Two patients were treated exclusively with radiotherapy because of non-resectability and poor general health.

#### No cancer treatment

Eight patients did not receive cancer treatment, mainly because of their poor condition; however, 19 patients (65.5%) needed some type of palliative procedure upon diagnosis or during the evolution of the disease (Table 4).

#### Effects of combined treatment

**Figure 2.**
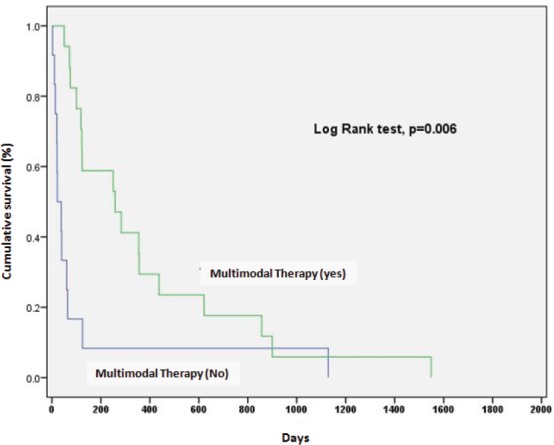
Impact of multimodal therapy in prognosis of anaplastic thyroid carcinoma.

A better survival rate was associated with combined treatment (surgery with radiotherapy, with or without systemic therapy) (*p* = 0.006) (Figure 2).

### Cause of death

26 patients died from ATC (93.1%): ten patients (34.5%) from locoregional disease, ten (34.5%) from locoregional and distant disease, and six from (20.7%) distant disease.

The specific causes of death were the following: lung disease, 14 patients (48.3%); respiratory failure due to obstruction to the airway, 16 patients (51.6%); malnutrition and cachexia, four patients (13.7%); and tumour bleeding, two patients (6.8%). However, several patients had concomitant causes of death.

Three patients (10.3%) died from diseases unrelated to the tumour. One patient, aged 81, clinical stage IVA with total resection, died from heart failure; another patient, aged 57, with a clinical stage IVB tumour and who received multimodal treatment, died from cardiac arrhythmia caused by acute ischemic cardiopathy.

### Survival

**Figure 3.**
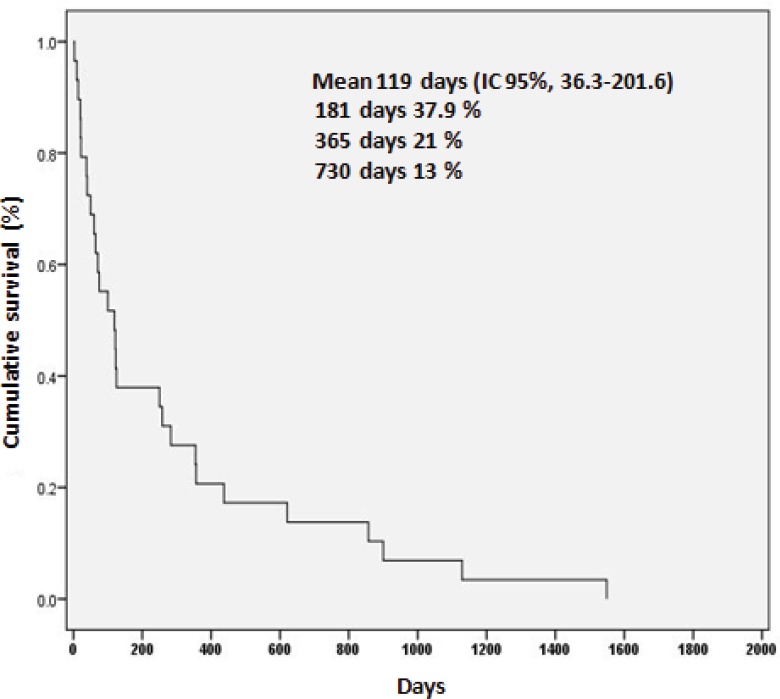
Survival of 29 patients with anaplastic thyroid carcinoma.

The average survival rate for the whole group was 119 days (IC 95%, 36.3–201.6). The survival rates at six months, one, and two years were 37.9%, 21%, and 13%, respectively (Figure 3).

**Figure 4.**
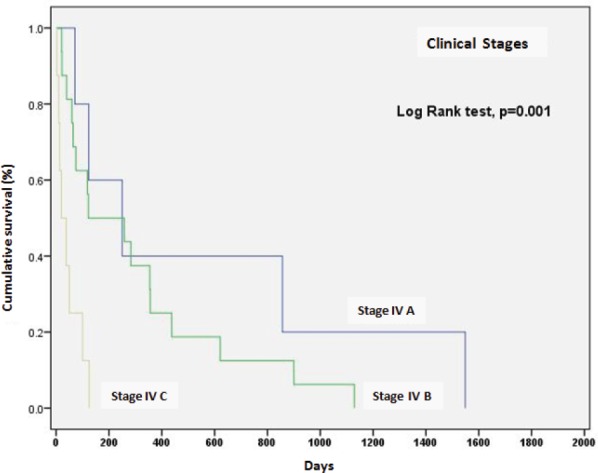
Survival according to clinical stage of anaplastic thyroid carcinoma.

The survival rate for clinical stages was 570 days for EC, IVA (IC 95%, 17.0–1122), 303 days for EC, IVB (IC 95%, 142–465), and 15 days for ECIV C (IC 95%, 13–76). The differences between clinical stages were statistically significant (*p* = 0.001) (Figure 4).

On the other hand, the global survival rate of EC IVA patients at six months was 59%, at a year 40%, at two years remained at 40%; for EC IVB, survival at six months was 51%, at a year 25%, and at two years 12.5%; for EC IVC, survival was 12% at 100 days and fell to 0% at 125 days.

The survival rate also correlated with the presence or absence of an extrathyroid disease. The best survival rate was found in patients with intrathyroid disease (*p* = 0.001).

## Discussion

ATC is a lethal disease, with specific mortality that reaches 100% [1]. Fortunately, it is a very rare neoplasm, and this has been corroborated by multiple authors. In the global scope, it is present in 1.3–9.8% of thyroid malignancies, associated with a survival median of five months and 20% in one year [12]. Akaishi *et al* found in 100 patients an overall survival median of 3.9 months (limits: 0.06–151.3); 40.4% of six months; 21.3% of a year, and 12.3% of two years [13]. Unfortunately, this makes it an obscure disease surrounded by controversy.

In our study, the survival median was 119 days with a lethality of 93%, which is similar to the reports of other authors; the same applies for the age. It has been suggested that at least half of the patients with anaplastic cancer would represent the dedifferentiation of a differentiated cancer. It is therefore not surprising that the age at which it is present is greater than that observed among patients with differentiated thyroid cancer in the same institution (46 versus 65 years of age). This suggests that it would take about 20 years to acquire and express mutations that transform the tumour to a more lethal variety [6–8, 14, 15].

There were multiple clinical manifestations. 23 patients (79.3%) reflected many functional implications of this fast-growing aggressive tumour. Tenvall *et al* pointed out that the manifestations tend to begin 1.5 months before hospitalization [9]. Manifestations due to local invasion seem more common in our study compared to what was indicated by Giuffrida *et al* [10]. This leads us to assume that we have a cohort with a disease in more advanced stages. However, the median of the reported tumour size oscillates between 6 and 9 cm [7, 8, 16, 17], and this is not different among our patients, since the median is 8 cm (5.5 ± 10.5). From our experience, at least 18 patients (62.1%) suffered from non-thyroid diseases (locoregional and distant metastasis) at the time of diagnosis. Other authors have pointed out that even in more than 90% of cases, regional and distant conditions could be present [18, 19].

In our study, 16 patients (55.2%) developed distant disease; similar to Venkatesh *et al* with (53%) [20]. We can summarise that our study was not significantly different when it comes to characteristics of the tumours and patients.

Us-Krasoveck *et al* reported 113 patients with ATC; Fine-needle aspiration biopsy (FNAB) produced a diagnosis of malignancy in 107 cases (94.7%), and 96 out of 107 were correctly diagnosed as anaplastic cancer [21]. Our study is different, in the sense that we only took FNAB samples from 10.3% of the patients. The majority (55.2%) initially had received incisional biopsies. We believe that it is necessary to have access to the tissue for the correct characterisation of the tumour, and we did not rely on FNAB since the suspicion often stems from clinical characteristics and is supported by tomographic imaging. With tissue, in obscure cases, it is possible to rely on, without limitation, the use of tumour markers (TTF1, PAX8, E-cadherin, p53, cytokeratin AE1/AE3) that allow ruling out tumours that require differential diagnosis, since they have a different prognosis and are treated in a different manner [21–23].

CT was the assessment of choice to evaluate locoregional extension of the disease. Specifically, eventual invasion of the aerodigestive tract, large cervical vessels, and structures of the mediastinum, as suggested by Takashima *et al* in this study of 13 patients (44.8%), the CT displayed unresectable criteria that modelled treatment [24].

Adequate treatment should increase the proportion of long-term survivors, while preserving the quality of life as much as possible. Without a doubt, surgery is the mode with the highest probability of achieving this objective, but it is still unsatisfactory, since a great majority of patients die from the disease, even when complete resection has been achieved. In addition, unresectability is not just a matter of technical capacity, it greatly depends on the extension of the disease in our opinion. This can be seen in this study consisting of 29 patients, of which 13 (44.8%) were not candidates for surgery, most of them because of clinical and radiologic findings compatible with unresectability. Only in nine patients (31%) was complete thyroidectomy possible, including a 45-year-old patient who had laryngopharyngectomy done in order to obtain complete tumour resection. In addition, two patients (6.8%) underwent central, anterolateral, and bilateral neck dissection, sacrificing the spinal nerve and internal jugular vein; however, in four out of 29 patients (13.7%), complete resection (R0) was achieved.

But even complete resection does not guarantee healing. ATC is not just a disease that is very aggressive at a local level; it also has a high potential for metastasis that is not avoided even with good local control. Let us recall that in our study, eight patients presented metastasis, and another eight developed it later. In the study from Raymond *et al* of 21 cases, complete resection was achieved in five patients, but he had survival of five years in 60%, and a median of survival of 131 months, but four of them received radiotherapy with or without concomitant chemotherapy; two of these patients survived for more than ten years, and the third patient survived for a few months without the disease in his follow-ups [18].

We are sure that complete resection is associated with the improved control of the disease and improvement in survival (*p* = 0.05). Although we should consider that complete resection is not responsible for improved survival, it is merely an indicator of a relatively small and confined thyroid disease, susceptible to resection. Therefore, in the majority of patients effective complementary therapy is required. Cheng *et al*, in an analysis of Surveillance, Epidemiology, and End Results program (SEER), demonstrated that the addition of post-operative radiotherapy was associated with improved survival when the disease spreads to adjacent tissue, in contrast to a confined disease for which no benefit was associated [25].

Our study confirms that radiotherapy, with or without chemotherapy, is associated with improved survival (*p* < 0.001), as shown in other cases [6, 26]. Even though McIver *et al* found that neither the extension nor the quality of resection affected survival (*p* > 0.04) [27], it is worth considering the best way to administrate it due to controversy. Wang *et al* in their retrospective study, compared hyperfractionated radiotherapy with radical doses in which a dosage greater than 40 Gy improved survival and local control, against palliative radiotherapy with dosage less than 40 Gy [28]. In our study, post-operative radiotherapy with dosage greater than the median of 33.1 Gy was associated with improved survival (*p* = 0.04).

Systemic therapy could also be linked to improved results [27]. Doxorubicin is the most widely used agent, as a sole radiosensitizing agent. In the randomized phase II study of Katsutaro *et al*, doxorubicin was compared as a simple agent in a group of 41 patients. Of those, seven patients (17%) presented partial responses; in the other group treated with doxorubicin and cisplatin, among 43 patients, a complete response was observed in five patients, and there was partial response in six patients, with a global response of 26%. However, of the five patients with complete response, four lived more than two years, compared to patients with partial responses who did not survive more than two years [29].

Doxorubicin, by itself or combined with platinum, appears to produce the best response rates and favourable medians of survival [1, 30, 32–34].

Surgery associated with chemotherapy and external radiotherapy seems to be the best treatment, since it at least prevents the obstruction of the airways [7, 10, 27]. This can be supported in our results, since multimodal treatment was associated with improved survival. In our research, from four patients who underwent complete resection, three received post-operative chemotherapy and radiotherapy concomitantly, and one received post-operative radiotherapy exclusively. Although the survival of this group was much greater than the rest of the group, the four patients eventually passed away from distant disease. Other uncontrolled studies seem to obtain the same conclusions [35–37].

Ken-ichi *et al* analysed 40 patients with ATC, treated between 1985 and 2009. They found that confined disease with low volume was associated to improved prognosis; however, they also corroborated improved results with combined treatment [38].

The expectancy of improved results has induced the use of therapies directed towards molecular targets. Sorafenib, an antineoplastic medication approved by the U.S. Food and Drug Administration (FDA) for advanced thyroid cancer, inhibits receptor tyrosine kinases, acting on receptors of angiogenic factors [39]. In a phase II study with 41 patients of metastatic papillary thyroid cancer, only six patients presented partial responses, and 23 patients presented stable responses, reporting median progression survival of approximately 15 months, making this a reasonable therapy that is well tolerated against the biological activity of metastatic papillary thyroid cancer [40]. As opposed to Savvides *et al*, also a phase II study of Sorafenib in ATC, with 20 patients evaluated, obtained partial responses in two patients (10%), and stable disease in five (20%), with a duration of the response of ten months in the two patients and 27 months in the patients who presented stable disease, with a median of four months (limits: 3–11 months), with a dosage of 400 mg twice a day [41].

## Conclusions

Our study has many limitations. It consists of a small group, subject to distinct treatments, which necessarily limits the capacity of our conclusions; however, it is possible to make some generalisations.

We managed to identify that complete surgical resection is one of the most important factors in the improvement of survival in contrast to incomplete resections. But we want to point out that complete resection reflects extension and behaviour of the tumour more than a technical capacity. Implementing multimodal therapy was associated with a favourable differentiation in terms of median of survival.

Finally, more efficient systemic treatments are needed, since very extensive resections do little to improve survival with regards to disease with a great capacity of locoregional and metastatic invasion.

## Conflicts of interest

We, the author and co-authors, declare that we have no personal, financial, or industrial conflicts of interest.

## Figures and Tables

**Table 1.** Clinical manifestations reported by 29 patients with anaplastic thyroid carcinoma.

Manifestations	Number of Patients (%)
Volume Increase	27(93.10)
Dysphonia	23(71.31)
Dyspnea	15(51.72)
Tumour Mass	13(44.82)
Dysphagia	10(34.48)
Vocal Cord Paralysis	7(24.13)
Weight loss	3(10.34)

**Table 2.** Distribution by clinical phases in 29 patients with anaplastic thyroid carcinoma.

Clinical Stages	Patients (%)
CS IV A	3(10.3%)
CS IV B	18(62.1%)
CS IV C	8(27.6%)
**T**	**Patients (%)**
T4a	3(10.3%)
T4b	26(89.7%)
**N**	**Patients (%)**
N0	11(37.9%)
N1a	5(17.2%)
N1b	13(44.8%)
**M**	**Patients (%)**
M0	21(72.4%)
M1	8(27.6%)

**Table 3.** Clinical characteristics, detail of treatment, and patient outcome.

Patients	Age	Tumour Size (cm)	Extension of the Disease	CS	Metastasis at Diagnosis	Surgery	Residual Disease	Therapy	Survival (Days)/Death(F)
1	42	7	locoregional	IVB	absent	Tt+RND	R0	RT+CTX	857/F
2	45	7	locoregional	IVB	absent	Tt+LFT	R0	RT+CTX	365/F
3	73	6	locoregional*	IVB	absent	Biopsy	R2	RT+CTX	119/F
4	68	4	locoregional	IVA	absent	Tt+RND	R1	RT+CTX	316/F
5	57	11	locoregional	IVB	absent	Biopsy	R2	RT+CTX	122/F
6	80	5	locoregional	IVB	absent	Tt	R2	RT	1059/F
7	60	10	distance	IVC	present	Ts	R2	RT+CTX	100/F
8	56	10	distance	IVC	present	Biopsy	R2	None	3/F
9	60	20	locoregional	IVB	absent	None	R2	None	59/F
10	58	6	locoregional	IVB	absent	Tt	R1	RT+CTX	283/F
11	55	10	distance	IVC	present	None	R2	None	14/F
12	55	5	locoregional	IVB	absent	Tt	R0	RT	123/F
13	69	5	locoregional	IVB	absent	Tt	R1	RT+CTX	355/F
14	65	12	locoregional	IVB	absent	None	R2	RT	60/F
15	57	6	distance	IVC	present	Ts+RND	R2	None	125/F
16	72	18	locoregional	IVB	absent	Ts	R2	RT	258/F
17	68	4	locoregional	IVB	absent	None	R2	RT	1129/F
18	71	15	locoregional	IVB	absent	Ts	R2	None	64/F
19	65	4	local	IVA	absent	Tt	R0	RT+CTX	1549/F
20	81	8	local	IVA	absent	Tt	R1	RT	71/F
21	75	10	distance	IVC	present	None	R2	None	38/F
22	61	20	locoregional	IVB	absent	None	R2	None	30/F
23	67	9	distance	IVC	present	None	R2	None	19/F
24	90	6	locoregional	IVB	absent	None	R2	RT+CTX	621/F
25	54	9	distance	IVC	present	None	R2	RT+CTX	50/F
26	59	10	distance	IVC	present	None	R2	None	44/F
27	82	4	locoregional	IVB	absent	None	R2	RT+CTX	437/F
28	91	20	locoregional	IVB	absent	None	R2	None	22/F
29	35	6	locoregional	IVB	absent	None	R2	RT+CTX	75/F

CS: Clinical Stage;

Tt: Total Thyroidectomy;

LFT: Laryngopharyngectomy;

RND: Radical Neck Dissection;

Ts: Subtotal Thyroidectomy;

RT: Radiotherapy;

CTX: Chemotherapy;

F: fatalities;

* locoregional from N1b.

**Table 4.** Palliative procedures.

Surgical Procedures	No. Patients (%)
Tracheostomy	8(27.60)
Gastrostomy	6(20.70)
Traqueostomy + Gastrostomy	4(13.80)
Pleural Drainage	1(3.40)
